# Supplementary material for: Predicted 3D model of the M protein of Porcine Epidemic Diarrhea Virus and analysis of its immunogenic potential
Source: PLoS One. 2022 Feb 9;17(2):e0263582. doi: 10.1371/journal.pone.0263582 (PMC8827446; doi:10.1371/journal.pone.0263582)
Supplement: S1 Fig — (PDF) [file pone.0263582.s001.pdf]

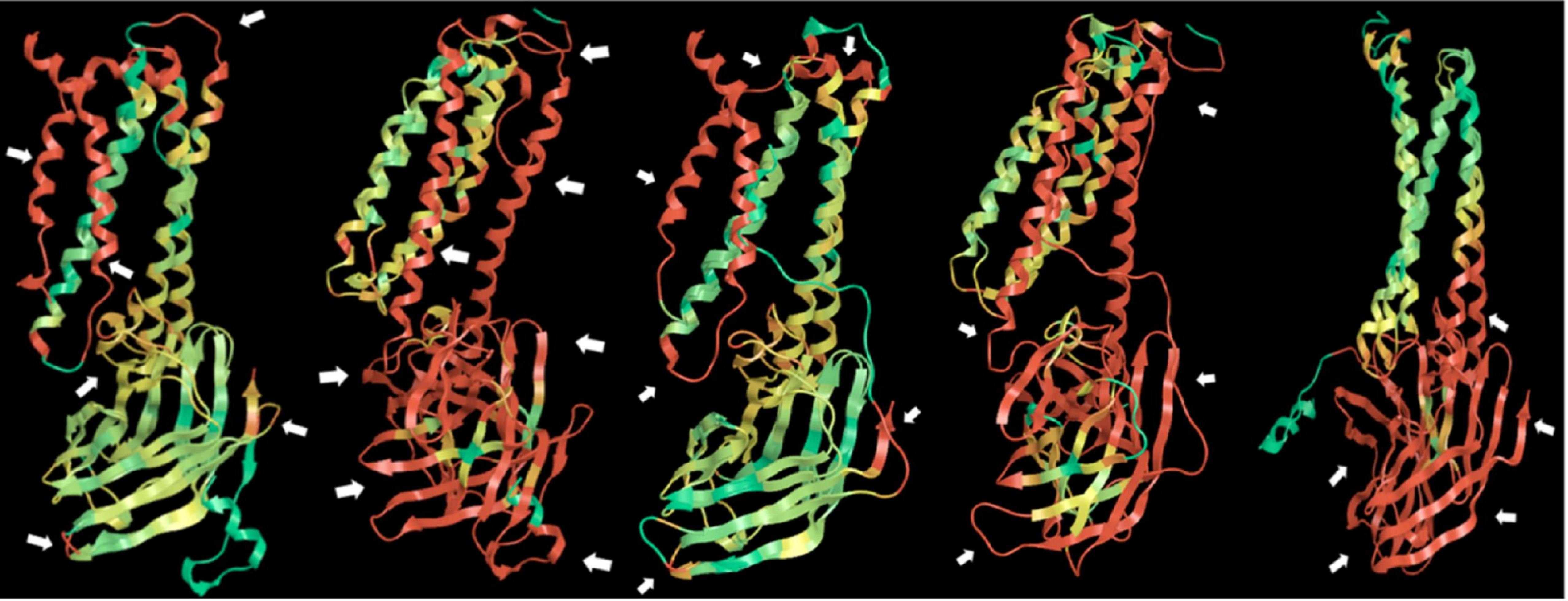

A B C D E

S1 Fig. Superimposition of the 3D M protein models from PEDV and SARS-CoV-2. A) Model of CV777 M protein and the SARS-CoV-2 M model from AlphaFold. B) Model of PEDV M protein CV777 strain and the SARS-CoV-2 M protein model from Feig lab. C) Model of 2013MMV M protein and the SARS-CoV-2 M protein model from AlphaFold. D) Model of 2013MMV M protein and the SARS-CoV-2 M model from Feig lab. E) Models of the SARS-CoV-2 M protein from AlphaFold and Feig lab. The colors refer to the RMSD value between the compared 3D models. If the RMSD value approaches 0 it is shown in green (there is no difference in predicting the 3D structure in that region of the protein). The RMSD value changes to yellow and orange as its value increases. The highest RMSD value and the largest difference between the predicted structures is represented in red. The white arrows indicate locally, the greatest changes in the spatial arrangement or in the type of predicted secondary structure.
