# Supplementary material for: Predicted 3D model of the M protein of Porcine Epidemic Diarrhea Virus and analysis of its immunogenic potential
Source: PLoS One. 2022 Feb 9;17(2):e0263582. doi: 10.1371/journal.pone.0263582 (PMC8827446; doi:10.1371/journal.pone.0263582)
Supplement: S1 Table — (PDF) [file pone.0263582.s002.pdf]

S1 Table. Comparison of the quality of the 3D models of the M proteins from PEDV and SARS-CoV-2. CV777 M protein and 2013MMV M protein constructed with Robetta with the 3D models of SARS-CoV-2 M protein obtained from AlphaFold and Feig lab.

**Program**	**CV777 Robetta**	**2013MMV Robetta**	**SARS-CoV-2 AlphaFold**	**SARS-CoV-2 Feig lab**
QMEAN	-1.58	-1.76	-0.86	-2.89
QMEANDisCo	0.59	0.57	0.49	0.45
PROCHECK*	89.2	88.1	94.8	88.6
MolProbity**	1.34 (2.21, 0.00, 95.09)	1.37 (3.05, 0.00, 95.96)	1.32 (1.57, 2.45, 97.38)	0.74 (0.28, 0.53, 96.36)
ProSA web***	-5.26	-5.88	-3.5	-3.87

* Percentage of angles in the most favored areas
** MolProbity value and the collision values of atoms, poor rotamers and angles in favored areas of the Ramachandran plot are shown in parentheses.
*** Z score values.
