# Supplementary material for: Predicted 3D model of the M protein of Porcine Epidemic Diarrhea Virus and analysis of its immunogenic potential
Source: PLoS One. 2022 Feb 9;17(2):e0263582. doi: 10.1371/journal.pone.0263582 (PMC8827446; doi:10.1371/journal.pone.0263582)
Supplement: S2 Table — (PDF) [file pone.0263582.s003.pdf]

S2 Table. Prediction of discontinuous epitopes with ElliPro in the 3D M protein models from PEDV and SARS-CoV-2.

CV777 M protein model			
**Epitope number**	**Residues**	**Number of residues**	**Score**
1	M1, S2, N3, G4, S5, I6, P7, V8, D9, E10, V11, I12, E13, H14, L15, R16, W18, N19, F20, T21, W22, N23, I24, L64, F65, D66, A67, W68, A69, S70, F71, Q72, V73, N74, W75, V76, F77, F78, A79, F80, S81, I82, L83, M84	45	0.741
2	A136, P137, T138, R200, S201, K202, H203, G204, D205, Y206, S207, A208, V209, S210, N211. P212, S213, A214, V215, L216, T217, D218, S219, E220, K221, V222, L223, H224, L225, V226	30	0.719
3	T27, I28, L29	3	0.698
4	V31, V32, Q29	3	0.684
5	S123, V124, M125, G126, R127, L144, L145, S146, G147, T148, A157, T158, G159, V160, Q161, V162, S163, Q164, L165, S186, V187, N188, A189, S190, S191, G192, T193	27	0.674
6	Y35, G36, H37, Y38, K39, Y40, S41, V42, F43, L44	10	0.645
**2013MMV M protein model**			
**Epitope number**	**Residues**	**Number of residues**	**Score**
1	I27, L28, V31	3	0.803
2	S1, N2, G3, F4, I5, P6, V7, D8, E9, V10, I11, Q12, H13, L14, R15, W17, N18, F19, T20, F64, D65, A66, W67, A68, S69, F70, Q71, V72, N73, W74, V75, F76, F77, A78, F79, L82, M83	37	0.747
3	H106, S122, V123, M124, G125, R126, Q127, G134, A136, P136, T137, G138, T142, L143, L144, S145, G146,T147, A156, T157, G158, V159, Q160, V161, S162, Q163, L164, P165, N166, F167, I177, V178, G180, R181, V182, G183, R184, S185, V186, N187, A188, S189, S190, G191, T192, G193,W194, A195, F196, V198, S200, K201, H202, G203, D 204, Y205, S206, A207, V208, S209.	60	0.699
4	L32, G35, Y37, K38, Y39, S40, A41, F42, L43.	9	0.548
**SARS-CoV- 2 M protein model from AlphaFold**			
**Epitope number**	**Residues**	**Number of residues**	**Score**
1	V143, I144, G147, H148, L149, H155, G157, R158, C159, D160, I161, K162, D163, L164, R186, V187, A188, G189, D190, S191, G192, F193	22	0.699
2	E12, L13, K14, K15, L16, L17, E18, Q19, W20, N21, L22, V23, I24, G25, F26, L27, T30, A63, C64, F65, L67, A68. A69, V70, Y71, R72, I73, N74, W75, T77, G78, G79, I80, A81, I82, A83, M84, C86	28	0.676
3	R101, A104, R105, E135, S136, E137, L138, V139, E167, S173, R174, T175, L176, S177, Y178, K180, L181, G182, A183, S184, Q185, A195, S197, R198, Y199, R200, I201, G202, N203	29	0.676
4	A40, N41, N43, R44, F45, L46, I49	7	0.573
**SARS-CoV- 2 M protein model from Feig lab**			
**Epitope number**	**Residues**	**Number of residues**	**Score**
1	S173, R174, K205, L206, N207, T208, D209, H210, S211, S212, S213, S214, D215, N216, I217, A218, L219, L220, V221, Q222	20	0.747
2	M1, A2, D3, S4, N5, G6, T7, I8, T9, V10, E11, E12, L13, K14, K15, L16. L17, E18. Q19, W20, V66, L67, A68, A69, V70, Y71, R72, I73, N74, W75, I76, T77, G78, G79, I80, A81, I82, A83, C86, L87, L90	41	0.739
3	I144, L145, R146, G147, H148, G157, R158, C159, D160, I161, K162, D163, L164, P165, K166, E167, K180, L181, G182, A183, S184, Q185, R186, V187, A188, D190, S191, G192, F193, A194	31	0.703
4	R107, L124, H125, G126, T127	5	0.686
5	Q36, F37, Y39, A40, N41, N43	6	0.665
