# Supplementary material for: Predicted 3D model of the M protein of Porcine Epidemic Diarrhea Virus and analysis of its immunogenic potential
Source: PLoS One. 2022 Feb 9;17(2):e0263582. doi: 10.1371/journal.pone.0263582 (PMC8827446; doi:10.1371/journal.pone.0263582)
Supplement: S3 Table — (PDF) [file pone.0263582.s004.pdf]

S3 Table. Prediction of discontinuous epitopes with DiscoTope in the 3D M protein models from PEDV and SARS-CoV-2.

CV777 M protein model					2013MMV M protein model				
Residue number	Residue name	Contact number	Propensity score	Discotope score	Residue number	Residue name	Contact number	Propensity score	Discotope score
5	SER	11	-0.488	-5.988	12	GLN	13	-0.452	-5.988
7	PRO	11	-0.269	-5.769	15	ARG	13	-0.242	-5.769
9	ASP	9	-0.726	-5.226	16	ASN	9	1.494	-5.226
10	GLU	11	-0.492	-5.992	17	TRP	12	-0.702	-5.992
13	GLU	12	-0.083	-6.083	18	ASN	12	-1.154	-6.083
20	PHE	12	-1.389	-7.389	39	TYR	10	-1.852	-7.389
37	HIS	10	-1.703	-6.703	103	ARG	12	1.41	-6.703
38	TYR	9	-1.208	-5.708	162	SER	9	-2.048	-5.708
104	ARG	13	0.851	-5.649	163	GLN	10	-2.42	-5.649
189	ALA	9	-2.024	-6.524	189	SER	8	-1.891	-6.524
190	SER	7	-1.6	-5.1	190	SER	8	-2.125	-5.1
191	SER	9	-2.805	-7.305	202	HIS	10	-0.463	-7.305
202	LYS	9	-0.019	-4.519	203	GLY	7	0.388	-4.519
203	HIS	9	0.273	-4.227	204	ASP	7	0.211	-4.227
205	ASP	14	-0.578	-7.578					
**SARS-CoV-2 M protein from AlphaFold Model**					**SARS-CoV-2 M protein from Feig lab Model**				
**Residue number**	**Residue name**	**Contact number**	**Propensity score**	**Discotope score**	**Residue number**	**Residue name**	**Contact number**	**Propensity score**	**Discotope score**
11	GLU	6	-0.824	-3.824	1	MET	12	-1.188	-7.188
12	GLU	8	-1.146	-5.146	3	ASP	9	-0.396	-4.896
13	LEU	9	-1.484	-5.984	4	SER	9	-0.396	-4.896
41	ASN	10	-0.801	-5.801	5	ASN	8	-0.133	-4.133
42	ARG	10	-0.801	-5.801	6	GLY	9	-0.396	-4.896
43	ASN	9	-0.694	-5.194	7	THR	12	-1.491	-7.491
45	PHE	10	-2.508	-7.508	42	ARG	10	-0.801	-5.801
126	GLY	8	-2.206	-6.206	43	ASB	11	-1.314	-6.814
162	LYS	11	-1.441	-6.941	115	GLU	14	1.057	-5.943
163	ASP	11	-1.052	-6.552	125	HIS	8	-2.206	-6.206
200	ARG	13	-0.069	-6.569	126	GLY	7	-1.762	-5.262
201	ILE	14	-0.161	-7.161	157	GLY	10	-2.308	-7.308
202	GLY	10	0.468	-4.532	163	ASP	8	-0.444	-4.444
203	ASN	8	0.669	-3.331	200	ARG	14	-0.345	-7.345
					201	ILE	15	0.429	-7.071
					202	GLY	13	1.418	-5.082
					203	ASN	13	1.329	-5.171
					204	TYR	12	0.106	-5.894
					205	LYS	13	-0.664	-7.164
					209	ASP	11	0.823	-4.677
					210	HIS	10	0.938	-4.062
					211	SER	10	0.938	-4.062
					212	SER	11	0.301	-5.199
					213	SER	10	-0.201	-6.201
