# Supplementary material for: Predicted 3D model of the M protein of Porcine Epidemic Diarrhea Virus and analysis of its immunogenic potential
Source: PLoS One. 2022 Feb 9;17(2):e0263582. doi: 10.1371/journal.pone.0263582 (PMC8827446; doi:10.1371/journal.pone.0263582)
Supplement: Attachment — Submitted filename: Response to Reviewers PONE-D-21-16531.pdf [file pone.0263582.s005.pdf]

Rebuttal letter

Dear Academic Editor's and reviewers

Below we present the changes made to the manuscript PONE-D-21-16531 Predicted 3D model of the M protein of Porcine Epidemic Diarrhea Virus and analysis of its immunogenic potential, according to your suggestions.

Academic Editor's comments:

Number	Comment	Answer
1	The referencing system is inaccurate and references numbers in the text do not match references listed in the bibliography. In the text, references are numbered in order of appearance, while in the list, references are numbered alphabetically. Thus numbers do not match. Please use the PLOS One recommended format from Instructions to authors: References are listed at the end of the manuscript and numbered in the order that they appear in the text. In the text, cite the reference number in square brackets (e.g., “We used the techniques developed by our colleagues [19] to analyze the data”).<br>When revising the references, please make sure the formatting is consistent. In particular the use of Title Capitalization is inconsistent (e.g .refs 11 and 27, maybe others)	Fixed the references section. The references are numbered in order of appearance in the manuscript and the format of each one was verified, according to the format recommended by PLOS One.
2	Legend of figure 1 contains unformatted references. Due to potential formatting issues, I do not recommend placing references in figure legends. It is better to provide the detailed methodological description in the material and methods section. In the legend, provide a simplified description of the approach and indicate “see material and methods for details and references”.	References and methodological description of legend 1 were removed and the legend “Details and references are provided in material and methods” was added.

3	There appears to be a discrepancy between the QMEAN values cited in the text (L131) and in Table 1. The text mentions -14.90 but the table mentions -14.60. Please correct or clarify.<br><br>There is another discrepancy of numbers between line 91 and 336 (percent identity). Please carefully revise numerical data since such discrepancies may negatively reflect on the study.	The correct value is -14.90 incorporated in the manuscript, it was corrected in the table. But attending the suggestion of reviewer 2 to shorten the manuscript and incorporate only the most relevant information. It was decided to delete the paragraph corresponding to the different programs that did not give good results, in which the aforementioned value is included.<br><br>The identity values 97.35-99.56% mentioned in line 91 were obtained by comparing the sequence of the M protein of the CV777 prototype strain with sequences from Europe, Asia and America (87 sequences), while the value that appears in line 336 corresponds to the comparison only of the M protein sequence of the 2013MMV strain excluding the CV777 strain. Since the second values are confusing, they were removed from the discussion section.
4	Ensure that figure legends are complete:<br><br>- Figure legend 1: indicate the significance of arrows. The text (L93) refers to three groups, clarify how these groups are identifiable from the tree.<br><br>- Figure legend 2: although panel C is described in a subsequent paragraph, the complete legend for figure 2 should be kept together (L189-192 added at L157)	The legends of figures 1 and 2 have been corrected. Also, the legends of figures 3, 4, 5 were modified to expose the meaning of arrows, colors and order of the information presented.
5	Ensure that all figures have a sufficient resolution so that they are easily readable when magnified.	The figures have been prepared according to the size, resolution and format indicated in the PloS One guide.
6	Typographic errors and minor changes:<br>L33: … virus (PEDV) is …<br><br>L38: interferon pathways<br><br>L91: 97.35-99.56%	 <br>The name of the virus was corrected.<br><br>pathways. It was corrected.<br><br>The values correspond to the maximum and minimum values of identity found between the

		compared amino acid sequences, referred to the M protein of strain CV777.
	L95: as a reference	It was included.
	L124: It is clear	It was corrected.
	L132: remove period before parenthesis	It was removed.
	Table S6: (Excl. Gly and Pro)	It was corrected Excl. Gly. But finally we decided to eliminate the table to include the most relevant information.
	Table S6: define asterisks	The table was corrected but eventually removed, due to the suggestion to include only the most relevant information.
	L146: It is mentioned that the most important template was ORF3. It's not clear how "importance" is defined and evaluated. Pleas clarify. If the different models had different weight in generating the models, it should be indicated in the table.	No, no structure used in modeling is more important than others. To the modelling Robetta uses different 3D structures deposited in the PDB that are obtained by the DALI server. SARS-CoV-2 ORF3 was the first 3D structure identified because the greatest structural similarity. The sentence: most important was wrong. It was corrected. That information was relocated to the material and methods.
	Figure S7A: for consistency use "Model 1 of 2013MMV M protein"	The table was corrected. But the figure S7 was deleted to shorter the manuscript.
	L207: reference needed for both approaches	References 36-38 were included.
	Figure S8: provide significance of arrows and colors in legend.	The colors represent the RMSD value between the compared 3D models. If the RMSD value approaches 0, it is displayed in green (there is no difference in predicting the 3D structure in that region of the protein). The RMSD value changes to yellow and orange as its value increases. The highest RMSD value and the largest difference between the predicted structures are represented in red. The white arrows locally indicate the largest changes in spatial arrangement or type of predicted secondary structure.

	L215: 33% amino acid sequence identity	amino acid sequence identity was corrected.
	Table S9: PROCHECK	The table was corrected. But the table S9 was deleted to shorter the manuscript.
	L243: B cell	It was corrected: the B cell peptides RSVNASSGTG
	Table S10: first Row: Parker, Conservation. Hyphenate or adjust column width.	It was corrected. The table S10 become to table 1.
	Table S10: 2013MMV first peptide. Position cannot be 110-112.	It was corrected. Right position are 110-118
	Figure 5: it would be helpful if the colored residues were identified on the models.	We decided not to mark the colored residues to avoid saturating the image with information. But a short drawing of the scoring scale in color was included, to explain that the highest score marks the epitope in red and the lowest score in orange.
	Table S13: Remove rows in Spanish for 0401 and 0801.	It was corrected. Table S13 became to Table 2.
	Table S13: the title of the table refers to epitopes of CV777, but is contains a number of epitopes unique to 2013MMV (*). Modify the title of the table so that it better reflects its content.	It was corrected. The title was modified.
	Table 3: it's not clear to me why there is a range of identity. Please clarify.	The range of identity is obtained from the comparison between the epitope analyzed with all sequences used in the study.
	L304: the data do not really address the "feasibility" od a candidate vaccine, rather, it suggest the potential of the M protein for the development of a candidate vaccine. Please clarify.	The manuscript was corrected. The study provides theoretical data on the potential of the M protein to be considered as an immunogen in the development of a new vaccine for the control of PEDV.

	Figure 6: since the first B cell epitope is colored in red inf figure s 4 and 5, it is confusing to color T cell epitope also in red in figure 6. Make sure that the two types of epitopes are clearly differentiated in this figure.	We agree, with the comment of the confusion on the location of the T cell epitopes, it was corrected but the decision was made to delete the figure to shorten the manuscript.
	Figure 6: for consistency and simplification, use the 2013MMV abbreviation rather than the full name	The figure was modified but also was removed to shorten the manuscript and present only the most relevant information.
	L319: practices	It was corrected.
	L320: triggers of the host's	It was corrected.
	L324: PEDV was unknown	It was corrected.
	L326: the potential regions inducing an immune response	It was corrected.
	L328: constructed	It was corrected.
	L333-335: unclear sentence. Please rephrase	It was corrected in the manuscript. To confirm that the amino acid sequences selected to obtain the 3D model represents to the M proteins of PEDV, a comparison was made with strains from different outbreaks around the world.
	L336: 99.11% identity<br><br>L336: the identity numbers are different from the same sentence in line 91. Please explain or correct.	The identity values of line 336 correspond to the comparison only of the M protein of the 2013MMV strain that has 8 amino acid changes with the sequences of different latitudes on the planet, excluding the CV777 strain. Since the second values generate confusion they were removed from the discussion section.
	L348: beta-strands	It was corrected.
	L349: residues in length	It was corrected.
	L358: remove reference labeled by author names	It was corrected.

	L362: … 218 host proteins [reference needed]	It was corrected.
	L363, L368, L372: [reference also needed for these statements]	The statements between L362 and 363 correspond to the same reference published in 2020. In the corrected references it corresponds to number 45.<br>It was corrected in the manuscript. The current reference for the first two statements corresponds to number 46 of the corrected manuscript. The reference no. 38 corresponds to the statement on line 372.
	L376: verify the link (space in the link suggest it is incorrect)****	It was corrected in the manuscript. The link was verified, it gives access to the information.
	L400: reference format [31] [32] should be [31-32]	It was corrected in the manuscript.
	L405: reference for Zhang paper.	It was corrected in the manuscript. Corresponds to reference 8.
	L406: “last figure of the article” unclear which article this refers to. The last figure of this manuscript does not offer a comparison with other epitopes. The discussion comparing the present data and previously known M protein epitopes must be detailed and clarified (L398-399 and L407).	We agree that the last figure does not offer a comparison with other epitopes, the purpose was to concentrate the information of the prediction made but already presented in the manuscript. It was decided to eliminate figure 6 and show that none of the programs used for the comparison identified 100% the WAFYVRS epitope reported in reference 8. Some programs partially detected the residues that form the epitope.
	L413: the docking data do not confirm that these epitopes can bind MHC. The data suggest that the epitope can bind MHC. Binding has to be demonstrated before being confirmed.	It was corrected. The docking analysis confirmed that the epitopes found could interact with the SLA-1: 0401 allele, which suggests that the cellular immune response could be complemented by the humoral response.<br>However, the docking section was removed. As the second reviewer pointed out, Autodock Vina was not designed for protein and peptide analysis, we did not find enough items to disprove their observation, so we decided to remove them.

	L416: do you mean “demonstrated by comparing different strains”? please clarify.	It was corrected in the manuscript.
	L420: B cell and T cell not hyphenated	It was corrected in the manuscript.
	L420: this sentence could be interpreted that the epitopes were identified functionally. Indicate that these are proposed epitopes identified in silico.	The manuscript was corrected to indicate that in silico identification of B and T cell epitopes was performed.
	L425: Unclear: “showed to be” appears incorrect. Do you mean “will be”?	It was corrected. The 3D model of the PEDV M protein generated in the present study could be a useful tool to carry out further research on the development of vaccines or drugs to control PED.
	L435: corresponds	It was corrected in the manuscript.

Journal requirements

Number	Comment	Answer
1	Please ensure that your manuscript meets PLOS ONE's style requirements, including those for file naming	It has been verified and corrected.
2	Please remove any funding-related text from the manuscript and let us know how you would like to update your Funding Statement.<br><br>Please include your amended statements within your cover letter; we will change the online submission form on your behalf.	The funding information was removed from the Acknowledgments section.<br><br>The amended statement has been included in the cover letter. I requested to modify my Financing Statement:<br>JFRB, IHC, NHRM<br>Grant number: FONSEC-SAGARPACONACYT 2017-06-292826<br>Consejo Nacional de Ciencia y Tecnología<br>https://www.conacyt.mx/Convocatorias-fondos-sectoriales-constituidos.html

		Alan Rodríguez Enríquez (BUAP: 219470378) was supported by CONACYT (2019-000037-02NACF).<br>NO, The funders had no role in study design, data collection and analysis, decision to publish, or preparation of the manuscript.
3	We note that you have included the phrase “data not shown” in your manuscript. if the data are not a core part of the research being presented in your study, we ask that you remove the phrase that refers to these data.	The phrase "data not shown" was removed from the entire manuscript.
4	Please review your reference list to ensure that it is complete and correct. Any changes to the reference list should be mentioned in the rebuttal letter that accompanies your revised manuscript.	Follow references were added:<br><br>1. Reveles-Félix S, Carreón-Nápoles R, Mendoza-Elvira S, Quintero-Ramírez V, García-Sánchez J, Martínez-Bautista R, et al. Emerging strains of porcine epidemic diarrhoea virus (PEDv) in Mexico. Transbound Emerg Dis. 2020;67(2):1035-1041. doi: 10.1111/tbed.13426.<br>2. Jung K, Saif LJ. Porcine epidemic diarrhea virus infection: etiology, epidemiology, pathogenesis and immunoprophylaxis. Vet J. 2015;204(2):134-43. doi: 10.1016/j.tvjl.2015.02.017.<br>3. Arndt AL, Larson BJ, Hogue BG. A conserved domain in the coronavirus membrane protein tail is important for virus assembly. J Virol. 2010;84(21):11418-28. doi: 10.1128/JVI.01131-10.<br>4. Armstrong J, Niemann H, Smeekens S, Rottier P, Warren G. Sequence and topology of a model intracellular membrane protein, E1 glycoprotein, from a coronavirus. Nature. 1984;308(5961):751-2. doi: 10.1038/308751a0.<br>5. Zhang Q, Ke H, Blikslager A, Fujita T, Yoo D. Type III interferon restriction by porcine epidemic diarrhea virus and the role of viral protein nsp1 in IRF1 Signaling. J Virol. 2018;92(4):e01677-17. doi: 10.1128/JVI.01677-17.

		6. Kumar S, Stecher G, Tamura K. MEGA7: Molecular evolutionary genetics analysis version 7.0 for bigger datasets. Mol Biol Evol. 2016;33(7):1870-4. doi: 10.1093/molbev/msw054.<br>7. Haas J, Roth S, Arnold K, Kiefer F, Schmidt T, Bordoli L, et al. The Protein Model Portal--a comprehensive resource for protein structure and model information. Database (Oxford). 2013;2013:bat031. doi: 10.1093/database/bat031.<br>8. Haas J, Gumienny R, Barbato A, Ackermann F, Tauriello G, Bertoni M, et al. Introducing "best single template" models as reference baseline for the Continuous Automated Model Evaluation (CAMEO). Proteins. 2019;87(12):1378-1387. doi: 10.1002/prot.25815.<br>9. Holm L. Using Dali for Protein Structure Comparison. Methods Mol Biol. 2020;2112:29-42. doi: 10.1007/978-1-0716-0270-6_3.<br>10. Lomize MA, Pogozheva ID, Joo H, Mosberg HI, Lomize AL. OPM database and PPM web server: resources for positioning of proteins in membranes. Nucleic Acids Res. 2012;40(Database issue):D370-6. doi: 10.1093/nar/gkr703.<br>11. Herráez A. Biomolecules in the computer: Jmol to the rescue. Biochem Mol Biol Educ. 2006;(4):255-61. doi: 10.1002/bmb.2006.494034042644.<br>12. Benkert P, Biasini M, Schwede T. Toward the estimation of the absolute quality of individual protein structure models. Bioinformatics. 2011;27(3):343-50. doi: 10.1093/bioinformatics/btq662.<br>13. Studer G, Rempfer C, Waterhouse AM, Gumienny R, Haas J, Schwede T. QMEANDisCo-distance constraints applied on model quality estimation.Bioinformatics. 2020;36(6):1765-1771. doi: 10.1093/bioinformatics/btz828.<br>14. Hodsdon ME, Ponder JW, Cistola DP. The NMR solution structure of intestinal fatty acid-binding protein complexed with palmitate: application of a novel distance

geometry algorithm. J Mol Biol. 1996;264(3):585-602. doi: 10.1006/jmbi.1996.0663.
15. Williams CJ, Headd JJ, Moriarty NW, Prisant MG, Videau LL, Deis LN, et al. MolProbity: more and better reference data for improved all-atom structure validation. Protein Sci. 2018;27(1):293-315. doi: 10.1002/pro.3330.
16. Wiederstein M, Sippl MJ. ProSA-web: interactive web service for the recognition of errors in three-dimensional structures of proteins. Nucleic Acids Res. 2007;35(Web Server issue):W407-10. doi: 10.1093/nar/gkm290.
17. Bui HH, Sidney J, Li W, Fusseder N, Sette A. Development of an epitope conservancy analysis tool to facilitate the design of epitope-based diagnostics and vaccines. BMC Bioinformatics. 2007;8:361. doi: 10.1186/1471-2105-8-361.
18. Haste Andersen P, Nielsen M, Lund O. Prediction of residues in discontinuous B-cell epitopes using protein 3D structures. Protein Sci. 2006;15(11):2558-67. doi: 10.1110/ps.062405906.
19. Ponomarenko J, Bui HH, Li W, Fusseder N, Bourne PE, Sette A, et al. ElliPro: a new structure-based tool for the prediction of antibody epitopes. BMC Bioinformatics. 2008;9:514. doi: 10.1186/1471-2105-9-514.
20. Zhang N, Qi J, Feng S, Gao F, Liu J, Pan X, et al. Crystal structure of swine major histocompatibility complex class I SLA-1 0401 and identification of 2009 pandemic swine-origin influenza A H1N1 virus cytotoxic T lymphocyte epitope peptides. J Virol. 2011;85(22):11709-24. doi: 10.1128/JVI.05040-11.
21. Jumper J, Tunyasuvunakool K, Kohli P, Hassabis D, the AlphaFold Team, “Computational predictions of protein structures associated with COVID-19”, Version 3, DeepMind website, 4 August 2020, https://deepmind.com/research/open-source/computational-predictions-of-

		protein-structures-associated-with-COVID-19<br>22. Huang YW, Dickerman AW, Piñeyro P, Li L, Fang L, Kiehne R, et al. Origin, evolution, and genotyping of emergent porcine epidemic diarrhea virus strains in the United States. mBio. 2013;4(5):e00737-13. doi: 10.1128/mBio.00737-13.<br>23. Kern DM, Sorum B, Mali SS, Hoel CM, Sridharan S, Remis JP, et al. Cryo-EM structure of SARS-CoV-2 ORF3a in lipid nanodiscs. Nat Struct Mol Biol. 2021;28(7):573-582. doi: 10.1038/s41594-021-00619-0.
		We requested the incorporation of an author who supported the review and modification process of this manuscript according to the suggestions of the reviewers and the academic editor. I present the application and the signature of the authors authorizing the modification for the incorporation of Lourdes Millán Pérez Peña.

Reviewer 1's comments

Number	Comment	Answer
**1**	Is there the M-gene knockout study to decipher the precise biological role of the M protein?<br><br>Any literature evidence to support that the M protein is a good therapeutic target?	In PEDV, the generation of the M protein knockout has not been reported. However, mutants have been generated in other M proteins of the Coronaviridae family to demonstrate their function. Although the M proteins of the coronavirus are different in amino acid sequence, they all share general structural characteristics. Until now it is known that the M protein of the coronavirus is the essential component for viral assembly and the release of virus-like particles. In the assembly of the virion, the M protein has interactions with the S protein, the N protein, but also lateral interactions between the M proteins, which are the main driving force for the formation of the viral envelope. [J Virol, 1998, 72:6838–6850; J Virol, 2000,74(11): 4967–4978; J Virol, 2010, 84(21):11418–11428; Virus Genes 2012, 44, 167–175]. VPLs are inducers of the production of neutralizing ACS against PEDV initially tested in mice [Virus Genes, 2017, 53: 548–554]. On the other hand, the

		function and importance of the M protein in the assembly of the virion has been confirmed through the generation of VLPs whose formation depends on the incorporation of the M, S and E proteins [Virus Genes, 2017, 53: 548–554]. In this work, it was confirmed that the E121R and E121K mutants of the M proteins did not form VPLs [J Virol, 2010, 84(21):11418–11428]. PEDV M protein also modulates the host immune response by inducing neutralizing antibodies in the presence of its complement [Virus Gene, 2012, 44(2):167-75], or by inhibiting IFN-β and interferón regulatory Factor 3 (IRF3) promoter activities [Virology, 2016 Feb; 489: 252–268.]. A high anti-PEDV-neutralizing antibody level was induced in mice vaccinated with VLPs and in pigs [Viruses 2020, 12, 1122]. In cultured swine intestinal epithelial cells, PEDV M protein induced cell cycle arrest at the S-phase via the cyclin A pathway [Acta virologica 59: 265 – 275, 2015]. Protein M is a key protein in the viral replication cycle of PEDV, therefore, it is feasible to consider it as a good therapeutic target.
2	Modeling the M protein in membrane.	We made the analysis. In the section Material and Methods, it was included the following: The 3D M protein models were placed in the membrane using the OPM (https://opm.phar.umich.edu/) by PPM server (http://opm.phar.umich.edu/server.php) to infer its spatial position in the membrane [20]. In the results it was included following: The spatial positions on the membrane of the 3D models of CV777 (Fig 2D) and 2013MMV M (Fig 2E) protein was analyzed with PPM server on OPM. The topology of both proteins is maintained, but the spatial orientation of the 2013MMV M protein changes in the extracellular region, in the arrangement of the α-helix on the transmembrane and globular regions.

Reviewer 2’s comments

Number	Comment	Answer

1	In general, the manuscript is difficult to read and should be reorganized and shortened. Only relevant information should be reported.<br><br>For example, several paragraphs in the Materials and methods can be merged.	The manuscript was abbreviated, to present only the most relevant information. The description of each procedure carried out was shortened in the materials and methods section. Figures and tables were removed from the results section. And the supporting information was shortened. We decided to relocate the material and methods section before the results.
	Major points:	
2	line 93: authors claim that three groups have been identified in the tree. This is not clear from Fig. 1 . Please, explain.	In the results section:<br>The M protein amino acid sequences were clustered into three groups related to G2a, G2b, G1 strains of PEDV. The figure 1 shows the 3 groups into which the sequences used in the study are grouped.<br>In discussion section:<br>The analysis included to the genogroups reported for the PEDV. There are different genogroups of PEDV, G1 and G2 (classic strain and variant strains), which can be divided into G1a and G1b, G2a and G2b. The G1a genogroup includes classic strains such as the CV777 prototype strain and other ancient PEDV strains. The G1b genogroup contains strains isolated in Asia with deletions in different genes, mainly in the genes encoding to the nsp3 and ORF3 proteins [39]. Genogroup G2 differs from G1 mainly due to amino acid changes in the NTD domain of protein S. Within the genogroup G2a there are circulating Asian strains reported in reemerging cases of PEDV and in the genogroup G2b there are circulating strains related to outbreaks in the United States and other parts of the world [40].
3	Line 107. ambiguous positions: not very clear. Please, explain	No, there are no ambiguous positions. The description is wrong, it refers to the gaps in the multiple alignment of amino acid sequences to build the phylogenetic tree. It was corrected in the manuscript.
4	Lines 112-126: all these data appear not strictly necessary. The paraghraph may be shortened	All information related to the path followed to find Robetta as the best option to build the 3D model of the M protein was deleted from the manuscript.

5	line 128: Please add a reference for the CAMEO project	The reference was included, it corresponds to: 18. Haas J, Gumienny R, Barbato A, Ackermann F, Tauriello G, Bertoni M, et al. Introducing "best single template" models as reference baseline for the Continuous Automated Model Evaluation (CAMEO). Proteins. 2019 Dec; 87 (12): 1378-1387. doi: 10.1002 / prot.25815. PMID: 31571280 PMCID: PMC8196401
6	line 138: it is not clear how the new PEDV sequence has been selected for modelling	2013MMV is a mexican strain isolated in 2013 in Michoacán, México; it has 6 amino acid differences compared to the CV777 M protein, is one of the strains isolated in cell culture that we keep under guard and that we have genetically characterized in its entirety by our research group.
7	line 149: The Table is not essential.	Table 1 was eliminated from the manuscript.
8	line 188. Fig. 2 legend is split. Is that correct?	The legend of figure 2 has been corrected.
9	line 194. Table 2 can be moved to the Supplementary data	Table 2 was eliminated from the manuscript, it provides a lot of information for only one of the programs, but the information from the evaluation of this program on the 3D models of protein M was kept in the manuscript.
10	line 287: Prediction of T-cell epitopes for the PEDV M protein: these methods are intrinsically inaccurate. Indeed, different prediction are obtained for different models of the same protein (Fig. 4), It may be interesting to show the consensus prediction.	NetMHCpan-4.1 server predicts peptide binding to any MHC molecule of known sequence using artificial neural networks. For this purpose, NetMHCpan-4.1 is trained by analyzing different combinations of peptides from a database of more than 850,000 ligand peptides identified by mass spectrometry and quantitative binding affinity. Based on this information, the server can make predictions for peptides of any length. We consider that the information presented in the table 2 in the current manuscript contains sufficient data to show the validity of the T cell epitope prediction. The consensus prediction was not included. However, we recognize that there are some limitations in the programs for the prediction of T epitopes, such as the redundant identification of peptides with variable sizes in the same region of the protein with multiple specificities towards the same epitope and the motifs of pig’s HLA class I have been few

		studied [PLoS Pathog. 2020 Jun 4;16(6):e1008607]. However, the program used is robust to give a reliable prediction.
11	Also, peptide docking is problematic. Indeed, Autodock Vina has not been specifically conceived for protein-peptide docking. Authors should explain better how they assigned binding energies (Table 3). A standard deviation should be calculated for each energy to account for the stocastic nature of the Vina algorithm. As a reference test, they should also calculate the binding energy of peptides not predicted to bind the SLA-01:0401 allele.	We consider the use of Autodock Vina because in our work group it is used routinely for the evaluation of the cellular and viral proteins - peptide interaction, but we agree that it was not developed to evaluate this type of interactions although it has been used in multiple research papers that have been published. Based on this information, and seeking arguments to justify the use of this program to assess the interaction of SLA-1: 0401 allele (PDB ID 3QQ3) with the 8 peptides identified with NetMHCpan. We did a molecular docking review with Autodock Vina. But this served to confirm the observation of reviewer 2 regarding the stochastic nature of the program. In the literature, the molecular docking of peptide ligands and proteins is not rigorously validated. It is known that there are limitations in the sampling algorithms, there is non-specificity of the coupling algorithms to predict the correct configuration of molecules with a high number of rotatable bonds such as peptides that are more flexible than proteins and adopt numerous conformations, the number of peptide residues influences the docking result and finally, limitations have been reported in the scoring functions that could influence the generation of a non-real interaction. [BMC Bioinformatics. 2019;19(Suppl 13):426.; Biochemistry. 2011; 50, 6157–6169.; J. Med. Chem. 2006; 49, 5912–5931.] Based on the evidence, we decided to eliminate the results obtained with AutoDockVina.
	Minor points	
12	Please, reformat carefully all references (for example, line 105)	The format of all references has been corrected.
13	Figure 3 should be moved to Supplementary data.	Figure 3 was removed from the manuscript to include only the most relevant information. Figure 3 only included information on a 3D structure validation method, therefore it was removed. However, the data obtained with the program were included in the manuscript.

We hope this manuscript is suitable for publication in your journal and we look forward to receiving your comments.

Sincerely,
The authors
