# Supplementary material for: Predicted 3D model of the M protein of Porcine Epidemic Diarrhea Virus and analysis of its immunogenic potential
Source: PLoS One. 2022 Feb 9;17(2):e0263582. doi: 10.1371/journal.pone.0263582 (PMC8827446; doi:10.1371/journal.pone.0263582)
Supplement: Attachment — Submitted filename: Response to Reviewers PONE-D-21-16531.docx [file pone.0263582.s006.docx]

**Rebuttal letter**

**Dear Academic Editor's and reviewers**

Below we present the changes made to the manuscript PONE-D-21-16531 Predicted 3D model of the M protein of Porcine Epidemic Diarrhea Virus and analysis of its immunogenic potential, according to your suggestions.

**Academic Editor's comments:**

| **Number** | **Comment** | **Answer** |
| --- | --- | --- |
|  | I came upon a number of typographic errors that must be corrected | All typographic errors were corrected. |
|  | I would strongly recommend that the manuscript should corrected by a native English speaker, to make the text more fluent, thereby increasing the impact of your data on the reader. | The manuscript was proofread by an English speaker. As proof, I attached the invoice dated 10/01/2022, from the document review service identified as (Cuartillas) Pages. |
|  | ***Specific editorial comments***  Title: update your title on manuscript title page. Currently it is duplicated with a second version, which seems to be the short title. Make sure both titles are separate to avoid confusion. | It was corrected. |
|  | The English language reviewer suggested changing the format of affiliations.  ^1^ Laboratorio de Bioquímica y Biología Molecular, Centro de Química, Instituto de Ciencias, Benemérita. Universidad Autónoma de Puebla, Puebla, México.  ^2^ Posgrado en Ciencias Químicas, Benemérita Universidad Autónoma de Puebla, Puebla, México.  ^3^ Laboratorio de Biología Molecular y Virología, Centro de Investigación Biomédica de Oriente, Instituto Mexicano del Seguro Social (IMSS), Metepec, México, México.  ^4^ Centro Nacional de Investigación Disciplinaria en Salud Animal e Inocuidad, Instituto Nacional de Investigaciones Forestales, Ciudad de México, México. | ^1^ Laboratory of Biochemistry and Molecular Biology, Chemistry Center, Institute of Science, Benemérita Universidad Autónoma de Puebla, Puebla, México.  ^2^ Postgraduate in Chemical Sciences, Benemérita Universidad Autónoma de Puebla, Puebla, México.  ^3^ Molecular Biology and Virology Laboratory, Centro de Investigación Biomédica de Oriente, Instituto Mexicano del Seguro Social (IMSS), Metepec, México, México.  ^4^ Centro Nacional de Investigación Disciplinaria en Salud Animal e Inocuidad, Instituto Nacional de Investigaciones Forestales, Ciudad de México, México. |
|  | L40 : remove “obtained” | L39. It was removed. |
|  | L55: replace “the virus” by “viral” | L54. It was replaced. |
|  | L64: add space before “and” | L63. It was applied. |
|  | L114: superposition should be superimposition | L114. It was replaced. |
|  | L166: “that we have genetically characterized in its entirety by our research group.” should be either “that we have genetically characterized in its entirety.” or “that was genetically characterized in its entirety by our research group” | L166-L667. It was replaced with the last suggested expression. |
|  | L173: replace “being” by “showing” or by “being the most similar” | L173. It was replaced by showing. |
|  | L174: M proteins | L174. It was corrected. |
|  | L185: The sentence is unclear. Change to: “Similarly, a value of depth/hydrophobic thickness of 20.8 ±1.9 Å, with a ΔGtransfer of -18.8 kcal/mol and tilt angle de 25±0 ° was calculated for the 3D 2013MMV M protein model.” | L185-L187. It was replaced. |
|  | L195: the figure legend for panel 2D mentions blue pseudo atoms when they are red in the figure. Also confirm that the color description of pseudoatoms in figure 2E is correct.  Also checks lines 365-367. | L197. Fixed mention of the color of pseudo atoms.  The mention of the color of pseudo atoms was verified in lines 366-368. |
|  | L205-206: avoid using square brackets [0.1] for values since they can be mistaken for references. “must be in a range between 0 and 1”. | L207. Brackets removed, “range [0.1]” was replaced by must be in a range between 0 and 1. |
|  | L236: replace “were compared finding” with “displayed” | L237. “were compared finding” was replaced with displayed. |
|  | S2 Table: there are no double ** in the table, but it is mentioned as a footnote. | Table S2: ** revised from footnote to table. The order of footnotes was checked. |
|  | L247: superimposition | L248. The word superimposing was replaced with superimposition. |
|  | L275: servers | L276. The word server was replaced with servers. |
|  | L287 and 296: Feig lab | L288 and L297. FeigLab was replaced with Feig lab. |
|  | L291: epitopes | L292. epitope was replaced with epitopes. |
|  | Table S3: last part of the table, epitope number 3: the word model is inserted in the sequence (near V70), it probably does not belong there. If it does, please explain. | Table S3: last part of the table, epitope number 3: the word model was eliminated. |
|  | Table S4: adjust width of fourth column so that “propensity” is on the same line. Otherwise, use proper hyphenation. | Table S4: Adjusted the width of the fourth column so that "propensity" is on the same line. |
|  | L328: structure of the M protein of PEDV is unknown | L329. “the 3D structure of the M protein of PEDV it was unknown” was replaced by structure of the M protein of PEDV is unknown. |
|  | L337: represent the M protein | L338. “represents to the M proteins” was replaced with represent the M protein. |
|  | L339: the analysis included the genogroups | L340. “The analysis included to the genogroups” was replaced by The analysis included the genogroups. |
|  | L353: remove space before “The” | L354. The space was corrected. |
|  | L354: change Fig2 to Figure 2 | L355. “Fig2” was replaced by figure 2. |
|  | L381: “can interact” instead of “interacts” since direct interactions with each cellular proteins were not directly demonstrated. | L380-381. “interacts” was replaced by can interact. |
|  | L395: period after [39] | L397. Period was added after [39]. |
|  | L397: allow making | L398-399. would allow to make was replaced by allow making. |
|  | L398: allow the design of drugs | L399-400. “allow to design drugs” changed by allow the design of drugs. |
|  | L401: replace “ the PEDV virus” with “PEDV” | L402. “PEDV virus” was replaced by PEDV. |

We hope this manuscript is suitable for publication in your journal and we look forward to receiving your comments.

Sincerely,

The authors
